# The 5th International Conference on Biomedical Engineering and Biotechnology (ICBEB 2016): Hangzhou, China. 1-8 August 2016

**DOI:** 10.1186/s12880-016-0164-6

**Published:** 2016-12-02

**Authors:** Shaoqing Wang, Xiancun Yang, Meixia Su, Qiang Liu, Tao Gong, Qi Mao, Shuguang Zhao, Fang Han, Keming Mao, Yixian Liu, Yanchun Zhu, Shuo Li, Jie Yang, Nan Fu, Shaode Yu, Rongmao Li, Jing Xiong, Yaoqin Xie, Shuihua Wang, Sidan Du, Zhimin Chen, Preetha Phillips, Shuwen Chen, Zeyuan Lu, Ping Sun, Zhengchao Dong, Yudong Zhang, Jingwen Zhuang, Junzheng Zheng, Mei Bai, Ning Mao, Xinnuan Mu, Cong Xu, Yulu Song, Xiaolei Song, Bin Wang, Haizhu Xie, Ke Gan, Daisheng Luo, Keming Mao, Zhuofu Deng, Jie Yang, Yanchun Zhu, Shuo Li, Nan Fu, Shaode Yu, Rongmao Li, Yaoqin Xie, Zhenghao Shi, Jiejue Ma, Minghua Zhao, Yonghong Liu, Yongchao Wang, Shuo Li, Yanchun Zhu, Jie Yang, Song Gao, Nan Fu, Shaode Yu, Yaoqin Xie, Yaping Wang, Guixue Liu, Wensheng Li, Changyu Tu, Lei Li, Ailong Cai, Linyuan Wang, Haibing Bu, Bin Yan, Junghua Ho, Yin Chang, Ioannis Manousakas, Chiehhsuan Wei, Xiaolong Sun, Juyoung Park, Soyeun Kim, Kyungtae Kang, Jingke Zhang, Feng Zhao, Guanyu Li, Yijie Ren, Yupei Chen, Xuming Zhang, Zhen Yu, Dong Ni, Siping Chen, Shengli Li, Tianfu Wang, Baiying Lei, Y. F. Li, Lanhua Zhang, Chengxin Yan, Huihui Yang, Baoliang Sun, Yanhui Ding, Yongxin Zhang, Yafeng Zhan, Tao Gong, Yuxiang Wu, Zhenghua Huang, Tianxu Zhang, Hao Fang, Yan Zhang, Zhijian Song, Manning Wang, Wan Li, Chunlan Yang, Feng Shi, Qun Wang, Shuicai Wu, Wangsheng Lu, Shaowu Li, Farnaz Farokhian, Yingnan Nie, Xin Zhang, Qingchun Li, Dongyan Yang, Yun Liang, Shihou Sheng, Xianbin Cheng, Baodong Gai, Binquan Li, Xiaohui Hu, Farnaz Farokhian, Chunlan Yang, Iman Beheshti, Hasan Demirel, Shuicai Wu, Wan Li, Yingnan Nie, Chunlan Yang, Qun Wang, Jiechuan Ren, Wan Li, Xin Zhang, Fuwen Lai, Mingwu Jin, Yifei Liu, Mingyue Ding, Yanhong Zhou, Huihong Gong, Wei Peng, Tao Gong, Wenyu Liang, Lili Zhao, Kuan Li, Jianping Yin, Mao Wang, Wei Liu, ZhiJun Gao, LiSha Tan, Ke Gan, Daisheng Luo, Shaoyin Duan, Simin Lin, Hua Zhong, Shaomao Lv, Haijun Lei, Jian Zhang, Zhang Yang, Baiying Lei

**Affiliations:** 1 Department of MRI, Shandong Medical Imaging Research Institute Affiliated to Shandong University, Jinan, Shandong 250021 People’s Republic of China; 2 Department of Interventional Radiology, Shandong Provincial Hospital Affiliated to Shandong University, Jinan, Shandong 250021 People’s Republic of China; 3 College of Information Science and Technology, Engr. Research Center of Digitized Textile & Fashion Tech. for Ministry of Education, Donghua University, Shanghai, 201620 China; 4 Intelligent multimedia information processing Lab, College of Software, Northeastern University, Shenyang, Liaoning Province 110004 China; 5 Institute of Biomedical and Health Engineering, Shenzhen Institutes of Advanced Technology, Chinese Academy of Sciences, Shenzhen, 518055 China; 6 School of Computer Science and Technology, Nanjing Normal University, Nanjing, China; 7 Department of Electrical Engineering, The City College of New York, CUNY, New York, USA; 8 Jiangsu Key Laboratory of 3D Printing Equipment and Manufacturing, Nanjing, China; 9 School of Electronic Science and Engineering, Nanjing University, Nanjing, Jiangsu, 210046 China; 10 College of Engineering, Nanyang Technological University, Singapore, 639798 Singapore; 11 School of Electronic Information, Shanghai Dianji University, Shanghai, China; 12 School of Natural Sciences and Mathematics, Shepherd University, Shepherdstown, WV 25443 USA; 13 Davis College of Agriculture, Natural Resources and Design, West Virginia University, Morgantown, WV 26505 USA; 14 State Key Laboratory of Millimeter Waves, Southeast University, Nanjing, 210096 China; 15 Center of Medical Physics and Technology, Hefei Institutes of Physical Science, Chinese Academy of Sciences, Hefei, China; 16 College of Agricultural and Life Sciences, University of Florida, Gainesville, FL 32611 USA; 17 Courant Institute of Mathematical Sciences, New York University, New York, NY 10012 USA; 18 Translational Imaging Division & MRI Unit, Columbia University and New York State Psychiatric Institute, New York, NY 10032 USA; 19 Guangxi Key Laboratory of Manufacturing System & Advanced Manufacturing Technology, Guilin, Guangxi 541004 China; 20 State Key Lab of CAD & CG, Zhejiang University, Hangzhou, 310027 China; 21 School of Computing, Mathematics and Digital Technology, Manchester Metropolitan University, Manchester, M156BH UK; 22 Department of Biomedical Engineering, Xuanwu Hospital of Capital Medical University, Beijing, 100053 China; 23 Department of Radiology, Yantai Yuhuangding Hospital, Yantai, Shandong 264000 People’s Republic of China; 24 Department of Radiology, Peking University People’s Hospital, Beijing, 100191 People’s Republic of China; 25 Department of Radiology, Binzhou Medical University Hospital, Binzhou, Shandong 256603 People’s Republic of China; 2600 Department of Nephrology, Yantai Chinese medicine hospital, Yantai, Shandong 264000 People’s Republic of China; 26 College of Electronics and Information Engineering, Sichuan University, Chengdu, China; 27 Intelligent multimedia information processing Lab, College of Software, Northeastern University, Shenyang, Liaoning Province 110004 China; 28 Institute of Biomedical and Health Engineering, Shenzhen Institutes of Advanced Technology, Chinese Academy of Sciences, Shenzhen, 518055 China; 29 School of Computer Science and Engineering, Xi’an University of Technology, Xi’an, 710048 China; 30 Xianyang Hospital, Yan’an University, Xianyang, 712000 China; 31 Medical Imaging Physics Laboratory, Peking University, Beijing, 100191 China; 32 Institute of Biomedical and Health Engineering, Shenzhen Institutes of Advanced Technology, Chinese Academy of Sciences, Shenzhen, 518055 China; 33 Department of Human Anatomy and Histoembryology, Shanghai Medical College, Fudan University, Shanghai, 200032 China; 34 Digital Medical Research Center, Shanghai of Basic Medical Sciences, Fudan University, Shanghai, 200032 China; 35 Shanghai Key Laboratory of Medical Imaging Computing and Computer Assisted Intervention, Shanghai, 200032 China; 36 Department of Ultrasound Diagnosis, Women and Children’s Hospital of Linyi, Linyi City, Shandong 276001 China; 37 National Digital Switching System Engineering & Technological Research Centre, Zhengzhou, 450002 Henan People’s Republic of China; 38 Department of Biomedical Engineering, National Yang-Ming University, 155, Sec.2, Li-Nong St., Peitou, Taipei, 11221 Taiwan; 39 Department of Biomedical Engineering, I-Shou University, Kaohsiung, Taiwan; 40 Department of Computer Science and Engineering, Hanyang University, Ansan, Republic of Korea; 41 School of Business Management, Hongik University, Sejong, Republic of Korea; 42 Department of Biomedical Engineering, School of Life Science and Technology, Huazhong University of Science and Technology, Wuhan, 430074 People’s Republic of China; 43 School of Biomedical Engineering, Shenzhen University, National-Regional Key Technology Engineering Laboratory for Medical Ultrasound, Guangdong Key Laboratory for Biomedical Measurements and Ultrasound Imaging, Shenzhen, China; 44 Department of Ultrasound, Affiliated Shenzhen Maternal and Child Healthcare, Hospital of Nanfang Medical University, Shenzhen, China; 45 Department of Information Engineering, Henan Polytechnic University, Jiaozuo, Henan 454000 People’s Republic of China; 46 School of Information and Engineering, Taishan Medical University, Taian, 271016 China; 47 Department of Medical Imaging, Affiliated Hospital of Taishan Medical University, Taian, 271000 China; 48 Key Lab of cerebral microcirculation in Universities of Shandong, Taishan Medical University, Taian, 271016 China; 49 School of Information Science and Engineering, Shandong Normal University, Jinan, China; 50 Brainnetome Center, Institute of Automation, Chinese Academy of Sciences, Beijing, China; 51 School of Mathematics Science, Shandong Normal University, Jinan, China; 52 School of Biomedical Engineering, Southern Medical University, Guangzhou, China; 53 College of Information Science and Technology, Engr. Research Center of Digitized Textile & Fashion Tech. for Ministry of Education, Donghua University, Shanghai, 201620 China; 54 Huazhong University of Science and Technology, Wuhan, Hubei 430074 China; 55 Wuhan Donghu University, Wuhan, Hubei 430074 China; 56 Digital Medical Research Center, School of Basic Medical Sciences, Fudan University, Shanghai, 200032 China; 57 Shanghai Key Laboratory of Medical Computing and Computer-Assisted Intervention, Shanghai, 200032 China; 58 School of Optical-Electrical and Computer Engineering, University of Shanghai for Science and Technology, Shanghai, 200093 China; 59 College of Life Science and Bioengineering, Beijing University of Technology, Beijing, China; 60 Biomedical Imaging Research Institute, Department of Biomedical Sciences, Cedars-Sinai Medical Center, Los Angeles, CA USA; 61 Department of Internal Neurology, Tiantan Hospital, Beijing, China; 62 Department of Internal Neurology, Puhua International Hospital, Beijing, China; 63 Department of Functional Neuroimaging, Neurosurgical Institute, Beijing, China; 64 Department of Gastrointestinal Surgery, China-Japan Union Hospital of Jilin University, Changchun, Jilin 130033 China; 65 Department of Ultrosond, China-Japan Union Hospital of Jilin University, Changchun, Jilin 130033 China; 66 Center of Physical Examination, China-Japan Union Hospital of Jilin University, Changchun, Jilin 130033 China; 67 School of Automation Science and Electrical Engineering, Beijing University of Aeronautics and Astronautics, Beijing, 100191 China; 68 The Institute of Software, Chinese Academy of Sciences, Beijing, 100190 China; 69 College of Life Science and Bioengineering, Beijing University of Technology, Beijing, 100022 China; 70 Integrative Brain Imaging Center, National Center of Neurology and Psychiatry 4-1-1, Ogawahigashi-cho, Kodaira, Tokyo, 187-8551 Japan; 71 Biomedical Image Processing Lab, Department of Electrical & Electronic Engineering, Eastern Mediterranean University, Gazimagusa, Mersin 10 Turkey; 72 College of Life Science and Bioengineering, Beijing University of Technology, Beijing, China; 73 Department of Internal Neurology, Tiantan Hospital, Beijing, China; 74 Department of Physics, University of Texas at Arlington, Arlington, TX 76051 USA; 75 School of Instrument and Electronics, North University of China, Taiyuan, Shanxi China; 76 School of Biomedical Engineering, Hubei University of Science and Technology, Xianning, Hubei 437100 China; 77 College of Life Science and Technology, “Image Processing and Intelligence Control” Key Laboratory of Education Ministry of China, Huazhong University of Science and Technology, Wuhan, 437400 China; 78 School of Medicine, Hubei University of Science and Technology, Xianning, Hubei 437100 China; 79 College of Information Science and Technology, Engr. Research Center of Digitized Textile & Fashion Tech. for Ministry of Education, Donghua University, Shanghai, 201620 China; 80 College of Computer, National University of Defense Technology, Changsha, Hunan 410073 China; 81 Institute of Software, College of Computer, National University of Defense Technology, Changsha, Hunan 410073 China; 82 State Key Laboratory of High Performance Computing, National University of Defense Technology, Changsha, Hunan 410073 China; 83 Network Center of Shenyang Jianzhu University, Liaoning, China; 84 Graduate School of Shenyang Jianzhu University, Liaoning, China; 85 Students’ Affairs Division of Shenyang Jianzhu University, Liaoning, China; 86 College of Electronics and Information Engineering, Sichuan University, Chengdu, China; 87 Medical Imaging Department, Zhongshan Hospital of Xiamen University, Xiamen, 361004 China; 88 College of Computer Science and Software Engineering, Shenzhen University, Key Laboratory of Service Computing and Applications, Guangdong Province Key Laboratory of Popular High Performance Computers, Shenzhen, China; 89 School of Information Engineering, Shenzhen University, Shenzhen, China; 90 School of Biomedical Engineering, Shenzhen University, National-Regional Key Technology Engineering Laboratory for Medical Ultrasound, Guangdong Key Laboratory for Biomedical Measurements and Ultrasound Imaging, Shenzhen, China

## 01 The application value of three-dimensional rotational angiography of intracranial micro-aneurysms in diagnosis and treatment

### Shaoqing Wang^1^, Xiancun Yang^2^, Meixia Su^1^, Qiang Liu^1^

#### ^1^Department of MRI, Shandong Medical Imaging Research Institute Affiliated to Shandong University, Jinan, Shandong, 250021, People's Republic of China; ^2^Department of Interventional Radiology, Shandong Provincial Hospital Affiliated to Shandong University, Jinan, Shandong, 250021, People's Republic of China

##### **Correspondence:** Qiang Liu (2002md@163.com)

**Aims**

To evaluate the diagnostic value of three- dimensional rotational angiography (3D-RA) of intracranial micro-aneurysms (diameter ≤ 3 mm) and provide guidance on the value of endovascular treatment.

**Materials and methods**

43 patients with intracranial micro-aneurysms were analyzed retrospectively, all patients had undergone angiography with both conventional 2D-DSA(Two-Dimensional Digital Subtraction Angiography) and rotational angiography with three-dimensional reconstruction; the frequency of detection of aneurysms, depiction of aneurysm neck, radiation dose, and the dosage of contrast agent were recorded respectively.

**Results**

55 pieces of aneurysms were detected out from the 43 cases with intracranial micro-aneurysms by 3D-RA. But only 39 cases were detected out using 2D-DSA from the 55 samples, there were significant differences with regards to detection rate (P < 0.05). There were significant differences in radiation dose and dosage of contrast agent (P < 0.05) between the two methods of using 3D-RA can improve the detection rate of micro-aneurysms, which bestows obvious advantages on displaying the shape of aneurysms, the aneurysm neck at the best angle, and the relationship with the parent artery, at the same time, the amount of contrast agent and radiation dose are reduced in 3D-RA compared to 2D-DSA.

**Keywords**

Three-dimensional rotational angiography, Intracranial micro-aneurysm, Three dimensional reconstruction

**Acknowledgments**

Funding: Shandong Natural Science Fund (Project No.Y2008C102)

Laboratory: Shandong Key Laboratory of Advanced Medical Imaging Technologies and Applications.

## 02 Recent advance of immunology-inspired medical imaging

### Tao Gong, Qi Mao, Shuguang Zhao, Fang Han

#### College of Information Science and Technology, Engr. Research Center of Digitized Textile & Fashion Tech. for Ministry of Education, Donghua University, Shanghai 201620, China

##### **Correspondence:** Tao Gong (taogong@dhu.edu.cn)

**Aims**

In order to improve the medical imaging, some immune computation theories and immune algorithms were reviewed and compared.

**Materials and methods**

The immune computation theories include the self and nonself theory, danger theory, artificial immune network etc. The immune algorithms include self/nonself detection algorithm, normal model construction algorithm, clonal selection algorithm, negative selection algorithm, danger model algorithm and hybrid immune algorithm etc. We improved the clonal selection algorithm to attain the optimal threshold for better segmentation of the medical images than the traditional approach.

**Results**

The X-ray medical image of the tuberculosis was processed with the improved clonal selection algorithm and noise filtering, and the output medical image of our approach is better for diagnosis than that of traditional image processing methods.

**Conclusions**

The immune algorithm can be improved to establish a better medical imaging, and this kind of medical application system is inspired from the human immune system.

**Acknowledgements**

Supported by the project grants from National Natural Science Foundation of China (Grand No. 61673007, 61271114, 11572084, 11472061 and 61203325), Natural Science Foundation of Shanghai (Grand No. 13ZR1400200), the Fundamental Research Funds for the Central Universities (DHU Distinguished Young Professor).

## 03 Medical image classification based on guided bagging

### Keming Mao, Yixian Liu

#### Intelligent multimedia information processing Lab, College of Software, Northeastern University, Shenyang, Liaoning Province, 110004, China

##### **Correspondence:** Keming Mao (maokm@mail.neu.edu.cn)

**Aims**

Traditional medical image classification methods focus on feature representation and classifier design. However, they seldom concerns data selection used for model training, which plays key role for model tuning and parameter optimization. This paper proposes a novel medical image classification method according to guided bagging.

**Materials and methods**

First, unsupervised learning is implemented for training image. Clusters are gained based on generative model. Then, at the discriminative model construction stage, training data is sampled covering all the data clusters, with a probability proportional to the density of each cluster. This method employs a well-distributed and balanced training data, and utilizes the virtue of generative and discriminative learning.

**Results**

The experiment uses the public available CT lung image dataset for evaluation. 379 lung CT images are contained, which are collected by 50 different CT lung scans. The standard data is described by the instruction of an expert. Experimental evaluations show that our proposed method has better performance in the field of lung nodule CT image classification comparing with traditional ones.

**Conclusions**

This paper utilizes the generative and discriminative training model, and a unified classifier is constructed for lung nodule classification. The proposed method is well-designed and the experimental results are preferable.

**Acknowledgements**

This paper was supported by National Natural Science Foundation of China (No. 61472073).

## 04 Cortical bone ultrashort TE study with inversion recovery preparation

### Yanchun Zhu, Shuo Li, Jie Yang, Nan Fu, Shaode Yu, Rongmao Li, Jing Xiong, Yaoqin Xie

#### Institute of Biomedical and Health Engineering, Shenzhen Institutes of Advanced Technology, Chinese Academy of Sciences, Shenzhen, 518055, China

##### **Correspondence:** Yaoqin Xie (yq.xie@siat.ac.cn)

**Aims**

Efficient improving contrast of cortical bone from surrounding long T2 tissues is important in ultarshort echo time (UTE) imaging.

**Methods**

UTE acquisition prepared by adiabatic inversion recovery were developed for this purpose. The effect of TI on cortical bone imaging was evaluated on mature bovine tibial mid-shafts using a 3-T clinical MR scanner. The imaging parameters were: TE/TR = 10 μs/300 ms, TI = 80, 90, 100, 110, 120, 130, and 140ms, FA =45°, Bandwidth = ±62.5 kHz, FOV = 8cm, slice thickness = 7mm, NEX = 2, single slice.

**Results**

With TI = 90ms, excellent suppression of long T2 signals was achieved with the CNR_cortical-muscle_ value of 13.49 ± 0.67, and the CNR_cortical-marrrow_ value of 12.26 ± 0.86. Due to different T1s of muscle and fat, some residual signals from fat were presented. Therefore, the CNR_marrow-muscle_ value was 1.24 ± 0.35. Furthermore, approximate 80% signals from muscle and fat were suppressed.

**Conclusions**

The 2D adiabatic inversion UTE sequence with a TI of 90 ms provided excellent contrast depiction of bovine cortical bone. Due to the T1 difference, muscle and fat longitudinal magnetizations cannot arrive to the null point at the same time. Therefore, simultaneous reduction of long T2 signals is complicated.

**Acknowledgements**

Supported by 81501463, 2014A030310360, 2011S013, 2015AA043203, JCYJ20140417113430639, SIAT Innovation Program for Excellent Young Researchers (201302), KQJSCX20160301144248, and Beijing Center for Mathematics and Information Interdisciplinary Sciences.

## 05 Preliminary research on brain tumor detection in MRI scanning based on wavelet entropy and kernel support vector machine trained by sequential minimal optimization

### Shuihua Wang^1,2,3^, Sidan Du^4^, Zhimin Chen^5,6^, Preetha Phillips^7,8^, Shuwen Chen^9^, Zeyuan Lu^10,11^, Ping Sun^2,12^, Zhengchao Dong^13,14^, Yudong Zhang^1,15,16^

#### ^1^School of Computer Science and Technology, Nanjing Normal University, Nanjing, China; ^2^Department of Electrical Engineering, The City College of New York, CUNY, New York, USA; ^3^Jiangsu Key Laboratory of 3D Printing Equipment and Manufacturing, Nanjing, China; ^4^School of Electronic Science and Engineering, Nanjing University, Nanjing, Jiangsu 210046, China; ^5^College of Engineering, Nanyang Technological University, Singapore 639798, Singapore; ^6^School of Electronic Information, Shanghai Dianji University, Shanghai, China; ^7^School of Natural Sciences and Mathematics, Shepherd University, Shepherdstown, WV 25443, USA; ^8^Davis College of Agriculture, Natural Resources and Design, West Virginia University, Morgantown, WV, 26505, USA; ^9^State Key Laboratory of Millimeter Waves, Southeast University, Nanjing 210096, China; ^10^Center of Medical Physics and Technology, Hefei Institutes of Physical Science, Chinese Academy of Sciences, Hefei, China; ^11^College of Agricultural and Life Sciences, University of Florida, Gainesville, FL 32611, USA; ^12^Courant Institute of Mathematical Sciences, New York University, New York, NY 10012, USA; ^13^Translational Imaging Division & MRI Unit, Columbia University and New York State Psychiatric Institute, New York, NY 10032, USA; ^14^Guangxi Key Laboratory of Manufacturing System & Advanced Manufacturing Technology, Guilin, Guangxi 541004, China; ^15^State Key Lab of CAD & CG, Zhejiang University, Hangzhou, 310027, China; ^16^School of Computing, Mathematics and Digital Technology, Manchester Metropolitan University, Manchester, M156BH, UK

##### **Correspondence:** Yudong Zhang (zhangyudong@njnu.edu.cn)

**Aims**

Brain tumors occur if abnormal cells form and accumulate within the brain. Two types of brain tumors exist as benign tumor and cancerous tumor. In order to detect brain tumors in MRI scanning in a more efficient way, we proposed a novel computer-aided diagnosis (CAD) system.

**Materials and methods:** A 100-image 256x256 T2-weighted MR brain dataset was obtained from the homepage of Harvard Medical School. Among the 100 images, 20 are normal control and 80 are with tumors. Our CAD system was established based on the hybridization of wavelet entropy (WE) and kernel support vector machine (KSVM). Our system firstly used WE to obtain distinguishing features from MR images on all subband coefficients obtained by discrete wavelet transform. 5-level Haar wavelet was utilized to obtain a sixteen-element vector.

The vector was fed into the classifier of KSVM that embedded kernel technique into plain support vector machine. The kernel was chosen as the radial basis function (RBF) function. We use grid-searching method to get the optimal RBF scaling factor as 1. KSVM was trained by sequential minimal optimization (SMO) algorithm.

**Results and Conclusion**

The 10 repetition of 10-fold stratified cross validation results showed the proposed WE + KSVM method achieved an excellent classification performance with an average accuracy of 98.80%, an average sensitivity of 99.50%, and an average specificity of 98.63%. The proposed “WE + KSVM” method is a promising brain tumor detection method for MRI scanning.

**Keywords**

brain tumor; detection; wavelet entropy; sequential minimal optimization; computer-aided diagnosis; radial basis function; cross validation; kernel support vector machine.

**Acknowledgements**

This paper was supported by NSFC (61602250, 61503188), Natural Science Foundation of Jiangsu Province (BK20150983), Jiangsu Key Laboratory of 3D Printing Equipment and Manufacturing (BM2013006), Program of Natural Science Research of Jiangsu Higher Education Institutions (15KJB470010), Special Funds for Scientific and Technological Achievement Transformation Project in Jiangsu Province (BA2013058), Open Fund of Guangxi Key Laboratory of Manufacturing System & Advanced Manufacturing Technology (15-140-30-008K), Open Project Program of the State Key Lab of CAD&CG, Zhejiang University (A1616), Open Fund of Key Laboratory of Statistical information technology and data mining, State Statistics Bureau, (SDL201608), Open Research Fund of Hunan Provincial Key Laboratory of Network Investigational Technology (2016WLZC013), and Open Fund of Fujian Provincial Key Laboratory of Data Intensive Computing (BD201607).

## 06 Study on geometric efficiency for MDCT

### Jingwen Zhuang, Junzheng Zheng, Mei Bai

#### Department of Biomedical Engineering, Xuanwu Hospital of Capital Medical University, Beijing, 100053,China.

##### **Correspondence:** Mei Bai (jswei65@163.com)

**Aims**

To investigate the dependence of geometric efficiency of a MDCT system on several exposure parameters such as tube voltage, collimation and pitch.

**Materials and methods**

Dose profiles in PMMA phantom for Siemens Definition Flash CT and GE Discovery CT750 HD were derived in helical mode using different tube voltages, collimations and pitches. Corresponding geometric efficiencies and weighted geometric efficiencies were calculated. Kruskal-Wallis test was performed to test the differences between weighted geometric efficiencies using different exposure parameters and the Spearman’s correlation coefficient was calculated to determine the correlation between different exposure parameters and weighted geometric efficiencies.

**Results**

With larger collimation the weighted geometric efficiency can be improved by 30%, while combined with larger pitch the weighted geometric efficiency can be reached to about 70%. Weighted geometric efficiencies had positive correlation with beam collimation and pitch (p < 0.05) for both CT scanners, while there was no significant difference between weighted geometric efficiencies with different tube potentials (p > 0.05).

**Conclusions**

The decrease of geometric efficiency leads to the increase of patient radiation dose. It is necessary to improve the geometric efficiency and reduce the burden of patients by optimal setting beam collimation and pitch for CT scans.

**Acknowledgements**

This work was supported by the National Natural Science Foundation of China (Grant No.81372923).

**Keywords**

multidetector computed tomography; geometric efficiency; radiation dose

## 07 Study of white matter in adolescent patients with depression by MR-diffusion tensor imaging

### Ning Mao^12#^, Xinnuan Mu^3#^, Cong Xu^4^, Yulu Song^3^, Xiaolei Song^3^, Bin Wang^3*^, Haizhu Xie^1*^

#### ^1^Department of Radiology, Yantai Yuhuangding Hospital, Yantai, Shandong, 264000, People’s Republic of China; ^2^Department of Radiology, Peking University People’s Hospital, Beijing, 100191, People’s Republic of China; ^3^Department of Radiology, Binzhou Medical University Hospital, Binzhou, Shandong, 256603, People’s Republic of China; ^4^Department of Nephrology, Yantai Chinese medicine hospital, Yantai, Shandong, 264000, People’s Republic of China

##### **Correspondence:** Bin Wang (binwang001@aliyun.com); Haizhu Xie (xhz000417@sina.com)

^#^ Ning Mao, Xinnuan Mu contribute equally to this work.

**Aims**

To explore the changes of the white matter in adolescent depression by using method of Tract-Based Spatial Statistcs (TBSS).

**Materials and methods**

We have applied TBSS to 35 depressed adolescents and 40 matched control to exam WM microstructure. With TBSS, we have concluded the fractional anisotropy (FA), axial diffusivity (AD), radical diffusivity (RD) and mean diffusivity (MD) of adolescent patients with depression and controls.

**Results**

Research found unusual WM structure among adolescent depression. Our analysis showed that the FA values are lower (P < 0.01), the RD and MD values are elevated (P < 0.01), and the AD values are Invariant (P > 0.05) in the patients’ body of the corpus callosum (CC). There is a contrary relationship between the severity of depression and FA values in the body of the CC(P < 0.01).

**Conclusion**

Our study showed that WM abnormalities are occurred in the pathophysiology of depression. What’s more, our research suggested that these changes occurred in the early stages of the disease.

**Keywords**

Adolescent Depression; diffusion tensor imaging; white matter

## 08 A landmark-based approach for mid-sagittal plane detection in 3D brain MR images

### Ke Gan, Daisheng Luo

#### College of Electronics and Information Engineering, Sichuan University, Chengdu, China

##### **Correspondence:** Ke Gan (gankeonline@hotmail.com)

**Aims**

This paper presents a fully automated approach for the mid-sagittal plane (MSP) detection in 3D brain MR images. This method detects the MSP by accurately identifying highly-visible anatomical landmarks in the brain.

**Materials and methods**

The proposed method is landmark-based, this involves a training phase, which is performed once for a particular set of data, using some spatially aligned images with known anatomical landmark locations. The center points of the anterior commissure (AC), posterior commissure (PC) and midbrain-pons junction (MPJ) were manually delineated on the training images by an expert. In the detecting phase, the intensity of the testing image was normalized and transform into the same space as the training images. The image feature of AC, PC, MPJ obtained in the training stage were used to match the AC, PC, MPJ in the testing image. To accelerate the matching, the landmark detection was conducted in the neighborhood of the mean AC, PC, MPJ positions in the normalized space. An refinement procedure was carried out to further adjust the detected landmarks. Finally, the formulation of the 3D MSP equation was estimated by the detected landmarks.

**Results**

The proposed method was applied to 30 T1-weighted brain MR images. All testing results were visually inspected and judged to be correct without obvious error. The directional difference of plane normal (DDPN) between automated detection and manual labeling has been evaluated, the average DDPN we achieved was 2.83°.

**Conclusions**

The promising results indicate this method can be potentially useful in clinical applications.

## 09 Medical image classification by multiple classifier learning

### Keming Mao, Zhuofu Deng

#### Intelligent multimedia information processing Lab, College of Software, Northeastern University, Shenyang, Liaoning Province, 110004, China

##### **Correspondence:** Keming Mao (maokm@mail.neu.edu.cn)

**Aims**

Medial image classification is a difficult task for its high similarity inter-class and low similarity intra-class. Traditional methods usually devise a single classifier. While, in this paper we focus on learning a multiple classifier for each type for medical image classification.

**Materials and methods**

The proposed method designs a boosted learning framework. First, an initial classifier is constructed according to the feature distribution of training image set. Then, more classifiers are trained in an iterative way. The overall performance can be enhanced successively. Moreover, the optimal weights can be gained on each individual classifier.

**Results**

The experiment uses the public available CT lung image dataset for evaluation. 379 lung CT images are contained in this dataset, which are collected by 50 different CT lung scans. The standard data is described by the instruction of an expert. Experimental evaluations show that the proposed method outperforms traditional methods with application to lung nodule CT image classification task.

**Conclusions**

This paper utilizes the boosted learning, combine multiple classifier for CT lung image classification. The proposed method exploits the feature representation distribution, and the experimental results are preferable.

**Acknowledgements**

This paper was supported by National Natural Science Foundation of China (No. 61472073).

## 10 Comparison between conventional and golden ratio based radial trajectories: an eddy currents study

### Jie Yang, Yanchun Zhu, Shuo Li, Nan Fu, Shaode Yu, Rongmao Li, Yaoqin Xie

#### Institute of Biomedical and Health Engineering, Shenzhen Institutes of Advanced Technology, Chinese Academy of Sciences, Shenzhen, 518055, China

##### **Correspondence:** Yanchun Zhu (yc.zhu@siat.ac.cn)

**Aims**

Golden ratio based k-space trajectories are widely used in dynamic MRI since it provides approximate uniform k-space distribution. However, rapidly switching gradient induces eddy currents, generating artifacts in image. The golden ratio (GR) based radial strategy was compared with conventional radial strategy on 0.7T open superconducting MRI system.

**Materials and methods**

In conventional radial strategy, a constant angle increment Φuniform = 180°/P between neighboring profiles. In GR based radial strategy, the azimuthal spacing is ΦGR = 180°/1.618 = 111.2°. First, a simulation was carried out to the comparison between ideal and net gradient of Shepp-Logan phantom with two radial strategies respectively. Second, a pure water phantom was sampled by Cartesian, conventional and GR based radial trajectory on the 0.7T open superconducting MRI system. Three orthogonal planes were acquired. SNR was compared between these three sampling trajectories, and images from Cartesian trajectory were used as reference.

**Results**

Result of simulation has illustrated that the impact of eddy currents on the ideal gradient with GR based radial strategy is more apparent. The result of a pure phantom shows that SNR values of both radial strategies (conventional: 40.76 GR:16.11) are far smaller than Cartesian strategy (145.15).

**Conclusions**

Eddy currents artifacts are more serious in GR based radial trajectory. Rapid switching gradient in GR based radial strategy induces more eddy currents than conventional radial strategy, which may limit the application of GR based trajectories especially in high magnet filed system.

**Acknowledgements**

Supported by 81501463, 81671853, 2014A030310360, 2011S013, JCYJ201500731154850923, KQCX20140521115045441, JCYJ20140417113430585, JCYJ20140417113430639.

## 11 ROI segmentation by localizing region-based active contours

### Zhenghao Shi^1^, Jiejue Ma^1^, Minghua Zhao^1^, Yonghong Liu^2^, Yongchao Wang^1^

#### ^1^School of Computer Science and Engineering, Xi’an University of Technology, Xi’an, 710048, China; ^2^Xianyang Hospital, Yan’an University, Xianyang, 712000, China

##### **Correspondence:** Zhenghao Shi (ylshi@xaut.edu.cn)

**Introduction**

Accurate segmentation of Region of interest (Region of Interest, ROI) has an important place in medical image analysis, and still remains a challenge task because of the complex background and structure.

**Materials and methods**

This paper proposed a novel active contour model based on localizing region for ROI segmentation in capsule endoscopy images. Features in regions centered on an active contour were used to compute the local region descriptors. For calculating the local energies, the image was separated by the initial circular shape curve into two parts: interior and exterior. And then each local region is fitted with a model to optimize the energies.

**Results**

Experiments show that in term of the average over segmentation rate, the proposed method is 2.8%, whereas traditional snake and GVF snake model are 3.2% and 3%, respectively; In term of the average under segmentation rate, the proposed method is 2.4%, whereas traditional snake and GVF snake model are 2.9% and 2.6%, respectively. All results demonstrated the superior of the proposed method to other existing methods in ROI segmentation.

**Acknowledgement**

This work is partially supported by the grant from the National Natural Science Foundation of China (No.61401355 and 61202198).

## 12 Accuracy and effectiveness of the respiratory self-gating signal in 3D cardiac cine MRI

### Shuo Li^1, 2^, Yanchun Zhu^2^, Jie Yang^2^, Song Gao^1^, Nan Fu^2^, Shaode Yu^2^, Yaoqin Xie^2^

#### ^1^Medical Imaging Physics Laboratory, Peking University, Beijing, 100191, China; ^2^Institute of Biomedical and Health Engineering, Shenzhen Institutes of Advanced Technology, Chinese Academy of Sciences, Shenzhen, 518055, China

##### **Correspondence:** Yaoqin Xie (yq.xie@siat.ac.cn)

**Aims**

Cardiac and respiratory self-gated free-breathing steady-state free procession (SSFP) has been proposed as an alternative to conventional SSFP for cardiac cine magnetic resonance imaging. In this technique, the acquired k-space data within the given respiratory gating windows are used for image reconstruction. Therefore, the accuracy of respiratory self-gating (RSG) signal is important.

**Materials and methods**

The self-gated free-breathing 3D SSFP technique was performed on a 1.5T GE HDx scanner. Twenty five healthy volunteers were included. The imaging parameters were: TR/TE = 3.5/1.3 ms, flip angle = 40°, bandwidth = ±125 kHz, slice thickness = 7 mm (no gap), number of slices = 12-14, number of profiles = 5000. RSG and respiratory bellow (RB) triggers were compared by correlation and t-test analyses. The percentage of respiratory signal intensity within gating windows was calculated.

**Results**

The respiratory cycle duration is 3314.7 ± 1072.6 ms. For all cases, the correlation coefficient between RSG and RB triggers is greater than 0.99, the P value of t-test is greater than 0.90. The percentage of RSG signal intensity within gating windows was 66.1 ± 4.1% compared with 60.1 ± 3.4% for RB.

**Conclusions**

There was an excellent correlation between RSG and RB triggers. There was no significant difference between two methods. RSG signal can well synchronize with RB signal and provide approximately the same respiratory cycle duration.

**Acknowledgements**

Supported by 2015AA043203, 81501463, 2011S013, 2014A030310360, JCYJ201500731154850923, JCYJ20130401170306812, JCYJ20140417113430585, JCYJ20140417113430639.

## 13 Changes in fiber bundles with aging: a tractography-based MRI study

### Yaping Wang^1,3^, Guixue Liu^1,3^, Wensheng Li^1,2,3^

#### ^1^Department of Human Anatomy and Histoembryology, Shanghai Medical College, Fudan University, Shanghai 200032, China; ^2^Digital Medical Research Center, Shanghai of Basic Medical Sciences, Fudan University, Shanghai 200032, China; ^3^Shanghai Key Laboratory of Medical Imaging Computing and Computer Assisted Intervention, Shanghai 200032, China

##### **Correspondence:** Wensheng Li (wshengli88@shmu.edu.cn)

**Aims**

The aim of this work was to investigate changes in fiber bundles with aging by utilizing quantified diffusion magnetic resonance imaging (MRI).

**Materials and methods**

A total of 125 normal subjects were separated into 5 groups (group 1: 16-30 years old, n = 20; group 2: 31-45 years old, n = 34; group 3: 46-60 years old, n = 24; group 4: 61-75 years old, n = 22; group 5: 76-90 years old, n = 25). All subjects underwent diffusion tensor imaging (DTI) and T1-weighted MRI in a 3T scanner, and DTI Studio software was used to process all DTI data and for tracing fiber bundles. Statistics for the total fiber number of brain and for the fiber density (FD) of 3 regions of interest (ROIs), namely the corpus callosum, cingulate, mesencephalon were gathered and analyzed using SPSS software.

**Results**

Significant differences were observed in total fiber number among all age groups (p < 0.05). In group 1, a significant difference was found between the FD of left and right cingulate (p < 0.05). Significant differences were found in comparisons of the FD of left and right cingulate (p < 0.05), and the downward trend of the left cingulate was found to be faster than that of right cingulate. Furthermore, significant differences were found between the FD of corpus callosum and cingulate (p < 0.05).

**Conclusions**

Thus, we can use quantitative MR DTI to study changes in brain fiber bundles.

**Acknowledgments**

This study was supported by the National Science and Technology Support Program (No.2015BAK31B01).

**Keywords**

diffusion tensor imaging; tractography; fiber bundle; aging

## 14 Ultrasonographic assessment of bony roof ratio in infant hip joints

### Changyu Tu (13969987078@163.com)

#### Department of Ultrasound Diagnosis, Women and Children’s Hospital of Linyi, Linyi City, Shandong 276001, China

**Aims**

This paper conducted research on a new method of ultrasound pediatric hip - bone top ratio measurement.

**Material and methods**

390 cases of pediatric hip (hip) ultrasound examination were selected since March 2011 to August 2016 according to Graf method of measuring the size of the angle α. They were divided into three groups: Group 1, α angle ≥60 °, 130 cases; Group 2, α angle <60 ° ~ ≥43 °, 130 cases; Group 3, α angle <43 °, 130 cases. On the basis of Graf law, the ratio of the top bone was measured.

We measure bony roof ratios in the following way. A tangent (referred as X axis) to the iliac bone was drawn across the transition point of iliac periosteum and perichondrium. A line vertical to X axis (referred as Y axis) was drawn across the vertex of acetabular cartilage roof. A vertical line to Y axis (line A) was drawn across the inferior boarder of acetabular labrum, and the other vertical line to Y axis was drawn from the inferior margin of ilium (the lowest point of ossificious iliac bone) (line B). The length of lines A and B was measured (cm) and the ratio between the length (A:B) was calculated as acetabular bony roof ratio (bony roof ratio).

**Results**

Group 1, α angle of 78.38 ° ~ 60.00 °, the top bone ratio 3.71 to 1.05; Group 2, α angle <60.00 ° ~ ≥43.00 °, the top bone ratio 1.21 to 0.54; Group 3, α angle <43.00 ° ~ 30.00 °, the top bone ratio from 0.58 to -0.36. Top bone ratio method classification criteria: I type, top-bone ratio > 1.2; II type, top-bone ratio of 1.2 to 0.6; eccentric, top bone ratio <0.6.

## 15 Fast primal-dual TV-based reconstruction and practical image-domain decomposition for few-view dual-energy CT

### Lei Li, Ailong Cai, Linyuan Wang, Haibing Bu, Bin Yan

#### National Digital Switching System Engineering & Technological Research Centre, Zhengzhou 450002, Henan, People's Republic of China

##### **Correspondence:** Bin Yan (ybspace@hotmail.com)

**Aims**

Dual-energy CT (DECT) has promising medical applications in differentiating bones and tissue. For full-view data, radiation dose is an important concern which makes few-view imaging research valuable. For few views, however, conventional filtered back-projection type methods fail to provide satisfying images, and direct decomposition is unstable due to noise boost. In order to obtain both high-quality CT and decomposition images for few views, this paper proposes a fast total variation (TV)-based image reconstruction algorithm and a practical image domain decomposition method for DECT.

**Materials and methods**

The reconstruction optimization problem, containing TV regularization term and data fidelity term, utilizes the pre-conditioned Chambolle-Pock method to design the algorithm which shows fast convergence. On the other hand, for noise suppression, the image decomposition uses the penalized weighted least square estimation, and applies the smoothness regularization term enforcing on estimation images. We implemented the proposed joint method on real DECT projections and compared it with state-of-the-art methods.

**Results**

The experiments on an anthropomorphic head phantom show that our methods have advantages on noise suppression and edge reservation, without blurring the fine structures in the sinus area. Furthermore, the proposed methods consume much less time than the compared iterative reconstruction algorithms.

**Conclusions**

This paper proposes a fast joint reconstruction-and-decomposition method for DECT imaging. Compared to the existing approaches, our method achieves better performance in reconstruction accuracy and decomposition quality.

**Keywords**

dual-energy CT imaging, primal-dual reconstruction, total variation regularization, image-domain decomposition

**Acknowledgements**

Supported by the National Natural Science Foundation of China (Grand No. 61372172).

## 16 A way to create a colored functional medical image by hardware

### Junghua Ho, Yin Chang

#### Department of Biomedical Engineering, National Yang-Ming University, 155, Sec.2, Li-Nong St.,Peitou, Taipei, 11221, Taiwan

##### **Correspondence:** Junghua Ho (kevinrho@ms5.hinet.net); Yin Chang (yichang@ym.edu.tw)

**Aims**

For many years, without software the medical imaging tools like X-ray, CT, Ultrasound, and MRI which only work in a monochromatic fashion. In order to display the colorful tissue image by hardware, we invented a color converter circuit to transfer the monochromatic tissue image into the color image to broaden the diagnostic and therapeutic possibilities.

**Materials and methods**

In order to colorize the monochromatic tissue images, the tissue reflection light is separated into the tissue image light and the bioluminescence fluorescence by a dichroic. Both lights are detected by two photo diodes and converted into the color signals by the color converter circuit.

**Results**

The two conditions of color changing on the color monitor are discussed as the following:(1) The black color on the monitor means there is no backscattered light coming from the tissue. (2) When there is fluorescent coming from the tissue, we can see the different color compounded showing on the color monitor.

**Conclusions**

By checking the color changed area on the color monitor to see the distribution of fluorescence on the tissue image, we can make the disease checking easier and clear.

## 17 Three dimensional ultrasound image analysis for renal calculi fragmentation monitoring during ESWL

### Ioannis Manousakas, Chiehhsuan Wei

#### Department of Biomedical Engineering, I-Shou University, Kaohsiung, Taiwan

##### **Correspondence:** Ioannis Manousakas (i.manousakas@ieee.org)

**Aims**

Up to now, little research has been done in the area of monitoring the fragmentation progress during Extracorporeal Shock Wave Lithotripsy (ESWL) treatment or renal stones while no method has been widely suggested for clinical practice. Experience of technicians and doctors are key factors for efficient treatments. In this study the use of 3D ultrasound imaging has been used for the estimation of the fragmentation level of renal stones during ESWL.

**Materials and methods**

Generally, in the course of lithotripsy, fragmentation of the stones produce changes in the intensity and texture of the images. First, simulated fragmented stones were used in a gelatin phantom. Furthermore, pig kidneys containing plaster of Paris stone models were exposed to shock waves. Gray level co-occurrence matrix texture features (contrast, entropy ASM, IDM and homogeneity) were calculated in 3D regions of interest containing the stones.

**Results**

The calculated texture values from the 3D regions containing the stone fragments show progressive changes which relate to the stone fragmentation level. The results from the gelatin phantom experiments show agreement with the pig kidney experiments.

**Conclusions**

Three dimensional ultrasound imaging could be used for monitoring of the progress of shock wave treatments of renal stones.

**Acknowledgements**

Supported by a project grant from MOST (MOST-103-2622-E-214-004-CC2).

## 18 Combining a support vector machine with a convolutional neural network for fMRI data classification

### Xiaolong Sun^1^, Juyoung Park^1^, Soyeun Kim^2^, Kyungtae Kang^1^

#### ^1^Department of Computer Science and Engineering, Hanyang University, Ansan, Republic of Korea; ^2^School of Business Management, Hongik University, Sejong, Republic of Korea

##### **Correspondence:** Kyungtae Kang (ktkang @hanyang.ac.kr)

**Aims**

Functional magnetic resonance imaging (fMRI) facilitates brain research and it is found to be widely used in clinic. Brain tasks can be related to regions of neural activity by classification of fMRI data. The design of previous systems has primarily been focused on classifier performance, whereas we focus on reliability.

**Materials and methods**

We use a hybrid system in which a convolutional neural network (CNN) is combined with a support vector machine (SVM). The CNN extracts features from the fMRI image and the SVM classifier finds patterns of features. Converting the dimension of the image and retaining its significant features increases processing speed and reduces the effect of noise, while the use of a simplified CNN reduces training time.

**Results**

Our system achieves a classification accuracy of 99.5% on Haxby's 2001 fMRI dataset, which is superior to decision tree, random forest, neural network, K-nearest neighbor, support vector machine, AdaBoost, and Haxby’s models. We contend that this makes it suitable for clinical applications.

**Conclusions**

Our hybrid system combines the advantages of SVMs and CNNs, which are both widely used for image recognition. The salient features extracted by our hybrid system do not have to be hand-coded, unlike those used by most existing classifiers. We found that the complexity of our hybrid model increased much less during the classification process than that of Haxby's model.

**Acknowledgements**

This work was supported by the Ministry of Science, ICT and Future Planning (MSIP), Korea, under the Information Technology Research Center (ITRC) support program (IITP-2016-H8501-16-1018) supervised by the Institute for Information & communications Technology Promotion (IITP), and by an IITP grant funded by the Korea government (MSIP; No. B0101-15-0557, Resilient Cyber-Physical Systems Research).

## 19 Spiking cortical model based structural representation for non-rigid multi-modal image registration

### Jingke Zhang, Feng Zhao, Guanyu Li, Yijie Ren,Yupei Chen, Xuming Zhang

#### Department of Biomedical Engineering, School of Life Science and Technology, Huazhong University of Science and Technology, Wuhan 430074, People's Republic of China

##### **Correspondence:** Xuming Zhang (zxmboshi@hust.edu.cn)

**Aims**

Structural representation based non-rigid multi-modal image registration (NMIR) methods have attracted much attention. However, many existing NMIR methods cannot provide satisfactory registration results. To address this problem, we have proposed a novel spiking cortical model (SCM) based structural representation method for the accurate NMIR.

**Materials and methods**

The reference and floating images are input into the SCM to generate the firing mapping images (FMI). The weighted mean of all the differences among Tchebichef moments of image patches centered at each considered pixel and other pixels in a neighbourhood in the FMI is used as a local descriptor to represent the image structure. The similarity metric is computed as the sum of squared differences between structural descriptors for the two images. By combining free-form deformation (FFD) with L-BFGS-B optimization method, the similarity metric is optimized to produce the registered image.

**Results**

Extensive experiments have performed on MR and CT images from BrainWeb database and Atlas database as well as ten real prostate MR and ultrasound images. Experimental results demonstrate that the proposed method can produce more similar registration results to the reference images and provide smaller target registration errors than the NMIR methods based on the normalized mutual information, entropy images, Weber local descriptor (WLD) and modality independent neighbourhood descriptor (MIND).

**Conclusions**

The proposed SCM based registration method provides an effective means for the accurate NMIR due to its robustness to image noise and rotational invariance.

**Acknowledgements**

Supported by project grants from the Fundamental Research Funds for the Central Universities (No.: 2015TS094) and Science and Technology Program of Wuhan, China (No.: 2015060101010027).

## 20 Automatic recognition of fetal facial standard plane using very deep convolutional neural network

### Zhen Yu^1^, Dong Ni^1^, Siping Chen^1^, Shengli Li^2^, Tianfu Wang^1^, Baiying Lei^1^

#### ^1^School of Biomedical Engineering, Shenzhen University, National-Regional Key Technology Engineering Laboratory for Medical Ultrasound, Guangdong Key Laboratory for Biomedical Measurements and Ultrasound Imaging, Shenzhen, China; ^2^Department of Ultrasound, Affiliated Shenzhen Maternal and Child Healthcare, Hospital of Nanfang Medical University, Shenzhen, China

##### **Correspondence:** Tianfu Wang (tfwang@szu.edu.cn); Baiying Lei (leiby@szu.edu.cn)

Ultrasound imaging has become a prevalent examination method in prenatal diagnosis. The most important precondition of routine US examination is the accurate acquisition of fetal standard plane from ultrasound videos. However, the labor-intensive and subjective assessment is too time-consuming and less reliability, and thus the development of the automatic fetal facial standard plane (FFSP) recognition method is urgently needed. In this paper, we propose to recognize FFSP using very deep CNN architecture (16 layer). In addition, a transfer learning strategy combine with special data augmentation technique is adopted to overcome overfitting problem and improves the classification performance. Also, a relatively shallower CNN architecture (8 layer) is exploited for FFSP recognition. The promising experimental results show the advantage of the proposed method vs the traditional manual features approaches, and indicate the effectiveness of deep CNN for detecting FFSP for clinical diagnose.

**Keywords:** Standard plane recognition; Ultrasound image; Deep CNN; Transfer learning.

## 21 Medical Imaging analysis based on Cloud Services Platform

### Y. F. Li (ppag200061b9a840@sohu.com)

#### Department of Information Engineering, Henan Polytechnic University, Jiaozuo, Henan, 454000, People's Republic of China

**Aims**

With the rapid development of technology, Cloud Computing turned out to be a reliable method to construct flexible and resilient regional medical imaging services platform. It will be cost-efficient and high-performance.

**Materials and methods**

By using various media with Cloud Computing, this paper describes the construction of regional medical imaging services platform and analyzes the needs of regional medical image sharing and cooperation and technology progress, and then designs a FC SAN and HDFS combination of medical imaging. Scalable distributed processing is the key to the platform. Storage regional services for SAAS based on the research goes for architecture and parallel medical imaging services.

**Results**

In consideration of the features of Hadoop, the requirement of medical imaging cloud platform and the basic structure of medical imaging, Could Computing is particularly designed for rapid large volumes of data (petabytes) storing and processing.

**Conclusions**

The reconstruction of medial cloud computing imaging services platform confronted great challenges in funds and technology with traditional technology. While newly-developed cloud computing and service model is a practicable approach to construct an economical, reliable and scalable regional medical imaging services platform.

## 22 Modelling and simulation of small world in memory network

### Lanhua Zhang^1*^, Chengxin Yan^2^, Huihui Yang^1^, Baoliang Sun^3*^

#### ^1^School of Information and Engineering, Taishan Medical University, Taian, 271016, China; ^2^Department of Medical Imaging, Affiliated Hospital of Taishan Medical University, Taian, 271000, China; ^3^Key Lab of cerebral microcirculation in Universities of Shandong, Taishan Medical University, Taian, 271016, China

##### **Correspondence:** Lanhua Zhang (acm_ict@163.com); Baoliang Sun (blsun88@163.com)

**Aims**

In order to model and simulate the small world topology in brain network on memory function, and explore the corresponding relationship between memory phenomena and functional characters.

**Materials and methods**

We design the deterministic algorithms to simulate the memory network of brain referring to the theories of graph, control and networks combing with the functional magnetic resonance imaging data.

**Results**

We simulate a memory network with evolution algorithms. By computing of network, the model has the small-world characters in clustering and average path length in accordance with the functional magnetic resonance imaging data results.

**Conclusions**

From computational model algorithm and memory phenomena, brain memory functional network also can be simulated with the same results of functional magnetic resonance imaging data. The method of cross subject research can provide a feasible way for the study of brain memory function network.

**Acknowledgements**

Supported by the Natural Science Foundation of Shandong (Grant No.ZR2013FL031), the Institutes of Higher Education Science and Technique Foundation of Shandong Province (Grant No.J15LE12).

## 23 Longitudinal analysis on altered functional connectivity in individuals at risk for Alzheimer’s disease

### Yanhui Ding^1,2^, Yongxin Zhang^3^ , Yafeng Zhan^2,4^

#### ^1^School of Information Science and Engineering, Shandong Normal University, Jinan, China; ^2^Brainnetome Center, Institute of Automation, Chinese Academy of Sciences, Beijing, China; ^3^School of Mathematics Science, Shandong Normal University, Jinan, China; ^4^School of Biomedical Engineering, Southern Medical University, Guangzhou, China

##### **Correspondence:** Yanhui Ding (yanhuiding@126.com)

Disruption of functional connectivity is increasingly considered to be associated with Alzheimer’s disease (AD) patient and that at high risk for AD. In this paper, altered functional connectivities are explored in longitudinal participants of 34 patients with EMCI, 23 patients with LMCI and 15 patients with AD, compared with 31 healthy control subjects. We evaluate the altered function connectivities with the progression of disease based on a priori defined 273 regions of interest.

Different levels of analysis based on functional connectivity are explored. Many inter- network connectivities and itra-network connectivities are found to be impaired in both MCI group and AD group. The longitudinal alterations of functional connectivity within DMN (Default Mode Network) were correlated with variation in cognitive ability, and the SAL (Salience Network) as well as the interaction between DMN and SAL was disrupted in MCI group. Importantly, the longitudinal alternation of functional connectivity in the earlier stage is greater than that in the late stage, and the increase of altered network connectivity pattern is associated with the increase of disease severity. The altered connectivities are correlated significantly with both MMSE scores and ADAS-Cog. This study indicates that altered connectivity might be a potential biomarker of AD progression.

**Acknowledgements:**

This work was supported by the Natural Science Foundation of China (No. 61303007).

## 24 Medical image segmentation with immunity-based improved fuzzy clustering algorithm

### Tao Gong, Yuxiang Wu

#### College of Information Science and Technology, Engr. Research Center of Digitized Textile & Fashion Tech. for Ministry of Education, Donghua University, Shanghai 201620, China

##### **Correspondence:** Tao Gong (taogong@dhu.edu.cn)

**Aims**

In order to optimize the initial center value for the medical image segmentation, an improved fuzzy clustering algorithm was proposed on the immune computation.

**Materials and methods**

The immunity-based improved fuzzy clustering algorithm searched the suitable initial center value quickly, increased the efficiency and accuracy, and avoided the blind local optimum. The antigen represented the grayscale image data, and the antibodies were generated at random to create the clusters. We designed a similar concentration factor and adjusted this parameter dynamically.

**Results**

The simulation results show that our improved algorithm can adaptively calculate the centers of the medical image clusters. The C-mean clustering algorithm used the initial cluster center (10, 80) and got the final threshold 103 in 6.1 seconds. Our proposed immune algorithm used the initial cluster center (65, 220), and got better final threshold 214 in 0.4 seconds.

**Conclusions**

We proposed an immune fuzzy clustering algorithm based on the improved preliminary optimum initial cluster centers, to avoid falling into the local minimization area and accelerate the searching speed. The lung image segmentation can be improved with our algorithm to help the doctors to analyze the lung disease better.

**Acknowledgements**

Supported by the project grants from National Natural Science Foundation of China (Grand No. 61673007, 61271114), Natural Science Foundation of Shanghai (Grand No. 13ZR1400200).

## 25 Sparse representation via adaptive dictionary for angiogram image denoising

### Zhenghua Huang^1^, Tianxu Zhang^1^, Hao Fang^2^

#### ^1^Huazhong University of Science and Technology, Wuhan, Hubei, 430074, China; ^2^Wuhan Donghu University, Wuhan, Hubei, 430074, China

##### **Correspondence:** Hao Fang (huangzhenghuahzh@163.com; 6974728@qq.com)

Image denoising is always an active research topic in the field of computer vision. The denoising performance had been greatly improved by the prior state-of-the-art methods based on non-local self-similar (NSS) patches. However, the prior NSS patch-based methods usually smoothed the edges and useful structures. The main reason is that the sparsity of the NSS patches cannot be correctly represented. In this paper, we propose a sparsely augmented Lagrangian image denoising (SALID) model over NSS patches via adaptive dictionary. With the adaptive dictionary, the sparsity of the NSS patches can be represented more efficiently. The results of widely synthetic experiments demonstrate that, with the single and effective alternating directions method of multipliers (ADMM) to solve the SALID model, the proposed denoising method can obtain highly competitive denoising performance and high-quality images, even superior to other advanced denoising methods. Moreover, the extensively experimental results of clinical X-ray angiogram images further verify that our method can obtain high-quality visual images.

**Keywords:** Angiogram image denoising; Non-local self-similar patch; Alternating directions method of multipliers

## 26 Structural and functional changes in patients with classical trigeminal neuralgia

### Yan Zhang^1,2,3^, Zhijian Song^1,2^, Manning Wang^1,2^

#### ^1^Digital Medical Research Center, School of Basic Medical Sciences, Fudan University, Shanghai 200032, China; ^2^Shanghai Key Laboratory of Medical Computing and Computer-Assisted Intervention, Shanghai 200032, China; ^3^School of Optical-Electrical and Computer Engineering, University of Shanghai for Science and Technology, Shanghai 200093, China

##### **Correspondence:** Zhijian Song ( zjsong@fudan.edu.cn)

**Aims**

In order to detect whether there are structural and functional changes of central nervous system in pain processing in classical trigeminal neuralgia (CTN) patients.

**Materials and methods**

Twenty-two patients with CTN and twenty-one age and sex-matched healthy volunteers were recruited. Whole brain 3D T1-weighted images and resting-state functional MRI datasets were obtained with SIMENS 3.0T MRI scanner. Voxel-based morphometry (VBM) based on DARTEL was used to identify the differences of gray matter volume and the Regional Homogeneity (ReHo) method was used to compare the brain spontaneous activity differences between patients and healthy controls.

**Results**

Compared with the health controls, the CTN patient presented with decreased GM volume in several brain regions including the right inferior temporal gyrus, inferior frontal gyrus, amygdala, thalamus, precuneus, cingulate gyrus and bilateral superior temporal gyrus, para-hippocampus, as well as increased GM volume in right frontal gyrus. Decreased ReHo values are in the left temporal and para-hippocampus, as well as increased ReHo values were noted in the bilateral thalamus and left parietal lobe between CTN patients and healthy controls.

**Conclusions**

CTN patients have multiple cerebral regions of GM volume abnormality and the abnormal spontaneous activity. These structural and functional abnormal regions are associated with the perception and processing of pain. All of these might reveal the exploration of central mechanisms of CTN.

**Keywords**

classical trigeminal neuralgia (CTN); Voxel-based morphometry (VBM); Regional Homogeneity (ReHo); Central nervous system

**Acknowledgments**

This study was supported by the National Science and Technology Support Program (No.2015BAK31B01).

## 27 What the original regional gray matter volume based cortical brain network can reveal of normal aging

### Wan Li^1^, Chunlan Yang^1*^, Feng Shi^2^, Qun Wang^3^, Shuicai Wu^1^, Wangsheng Lu^4^, Shaowu Li^5^, Farnaz Farokhian^1^, Yingnan Nie^1^, Xin Zhang^1^

#### ^1^College of Life Science and Bioengineering, Beijing University of Technology, Beijing, China; ^2^Biomedical Imaging Research Institute, Department of Biomedical Sciences, Cedars-Sinai Medical Center, Los Angeles, CA, USA; ^3^Department of Internal Neurology, Tiantan Hospital, Beijing, China; ^4^Department of Internal Neurology, Puhua International Hospital, Beijing, China; ^5^Department of Functional Neuroimaging, Neurosurgical Institute, Beijing, China

##### **Correspondence:** Chunlan Yang (clyang@bjut.edu.cn)

**Aims**

This study seeks to determine what alterations of the cortical brain network constructed using original regional gray matter volume (ORGMV) can be revealed for normal aging.

**Materials and methods**

The subjects are acquired from OASIS database. IBASPM toolkit was used to perform ORGMV measurement. Pearson correlation was used to build the network after linear regression. Network analysis was done by BCT. The attributes are interregional correlations, small-world configurations, nodal and modular properties. Lastly, statistical analysis was applied to verify the significant alterations.

**Results**

1) Decreased interregional correlation was found between the superior temporal pole and middle temporal pole in the right hemisphere, whereas increased cases occurred mostly in the frontal lobe between bilateral regions. 2) The distributions of hubs exhibited left-lateralized and right-lateralized in the young and aging group, separately. The fusiform gyrus and Rolandic operculum were identified as hubs for the young and aging groups, respectively. 3) Only one connector-module was found in the aging group, and the inter-module connections of one module in the aging group is relatively sparse.

**Conclusions**

To our knowledge, this study is the first to realize brain network construction by ORGMV. The findings suggest that it could enhance the understanding of the underlying physiology of normal aging and serve as a supplement approach to help exploring the mechanism of the human brain.

**Acknowledgements**

Supported by project grants from Beijing Nova Program (xx2016120), National Natural Science Foundation of China (81101107), Natural Science Foundation of Beijing (4162008), and Young Innovative Talents of the Beijing Educational Committee (CIT & TCD201404053).

## 28 The advantages of ultrasound in the treatment of abdominal malignant tumors by 125-iodine seed implantation

### Qingchun Li^1^, Dongyan Yang^2^, Yun Liang^3^, Shihou Sheng^1^, Xianbin Cheng^1^, Baodong Gai^1^

#### ^1^Department of Gastrointestinal Surgery, China-Japan Union Hospital of Jilin University, Changchun,Jilin, 130033, China; ^2^Department of Ultrosond, China-Japan Union Hospital of Jilin University, Changchun, Jilin, 130033,China; ^3^Center of Physical Examination, China-Japan Union Hospital of Jilin University, Changchun, Jilin, 130033,China

##### **Correspondence:** Baodong Gai (youth3003@163.com)

**Aims**

Abdominal viscera have its own particularity, so the choice of imaging guidance shows more importance in the process of 125-Iodine seed implantation. The goal of this study was to classify the advantages of ultrasound in the treatment of abdominal malignant tumors by 125-Iodine seed implantation.

**Materials and methods**

20 patients were evaluated in this study and they accepted 125-Iodine seed implantation guided by ultrasound. This paper aims to analyze the ultrasound guidance influence factors and measures and make clear its advantages.

**Results**

Among all 20 patients, 4 cases were liver malignant tumors. General anesthesia combined with breathing controlled could reduce the activity of liver, achieve fast and accurate punctures and the 4 cases had no serious complications; 7 cases were pancreatic cancer. Using ultrasonic probe to squeeze the stomach could reduce or avoid injury caused by multiple punctures, and the 7 cases had no gastric bleeding and fistula; 9 cases were retroperitoneal tumors. Using ultrasonic probe to press abdomen could reduce or avoid bowel and mesentery injury, gastrointestinal hemorrhage and the rates of intestinal fistula and abdominal infection, and the 9 cases had no melena; Real-time guidance was equivalent to direct vision, the puncture process could be monitored and showed its security.

**Conclusion**

This study found that ultrasound guided 125-Iodine seed implantation in the treatment of abdominal malignant tumors could achieve accurate puncture, reduce the puncture injury safely and effectively.

**Keywords**

Ultrosound, Radioactive seed implantation, 125-Iodine, Abdominal malignant tumor

## 29 Medical images classification using Mapreduce based convolutional neural network and its application for big data processing

### Binquan Li^1^, Xiaohui Hu^2^

#### ^1^School of Automation Science and Electrical Engineering, Beijing University of Aeronautics and Astronautics, Beijing, 100191, China

^2^The Institute of Software, Chinese Academy of Sciences, Beijing, 100190, China

##### **Correspondence:** Binquan Li (jz05022300@sina.com)

**Aims**

With the arrival of big data stage, big data analytics has become new challenge. High resolution computed tomography (HRCT) is major source of medical big data. HRCT classification is widely used in medical imaging missions. Motivated by the success of convolutional neural networks (CNN), we combine deep learning and big data methodology together to address the challenge.

**Materials and methods**

We take advantage of distributed computing and design the system on clusters. Different from traditional approach using single machine, we propose Mapreduce based CNN (MRCNN) significantly increasing training speed and reducing computation cost simultaneously. The feature vector is sent to Naïve Bayes classifier (NBC) for classifying interstitial lung disease and other medical HRCT. To avoid overfitting and local minima, we utilize genetic algorithm and Bayesian regularization (GABR) pre-training networks and initializing the weights. Furthermore, we design Mapreduce based NBC to increase efficiency of training classifier.

**Results**

Compared with other methods in past years, our method achieves benchmark performance. It’s capable of enhancing recognition accuracy and suitable for medical imagery big data processing.

**Conclusions**

MRCNN is efficient for storage and processing of HRCT big data. Due to the powerful feature representation ability of CNN, distributed MRCNN framework can be applied to other medical imaging big data analytics.

**Acknowledgements**

Supported by a project grant from National Natural Science Foundation of China (Grand No. U1435220).

## 30 Hippocampus volume atrophy in Alzheimer’s disease base on sex

### Farnaz Farokhian^1^, Chunlan Yang^1^, Iman Beheshti^2^, Hasan Demirel^3^, Shuicai Wu^1*^, Wan Li^1^

#### ^1^College of Life Science and Bioengineering, Beijing University of Technology, Beijing, 100022, China; ^2^Integrative Brain Imaging Center, National Center of Neurology and Psychiatry 4-1-1, Ogawahigashi-cho, Kodaira, Tokyo 187-8551, Japan; ^3^Biomedical Image Processing Lab, Department of Electrical & Electronic Engineering, Eastern Mediterranean University, Gazimagusa, Mersin 10, Turkey

##### **Correspondence:** Shuicai Wu (wushuicai@bjut.edu.cn)

**Aims**

Hippocampus, which is critical for memory, learning and declaration of emotional behaviors plays important role in Alzheimer’s disease (AD). We investigated hippocampus gray matter atrophy using analysis of variance (ANOVA) on 3-Tesla 3D T1weighted magnetic resonance imaging (MRI) data among four groups of participants.

**Materials and methods**

The experiments included 68 patients with AD and 68 normal control (NC) from ADNI database. They were divided into four groups (34 male patients with AD, 34 age-matched male NC, 34 female patients with AD and 34 age-matched female NC). Data processing was performed using Statistical Parameter Mapping (SPM8) and the VBM toolbox with defult setting. All structural MRIs were bias-corrected, segmented into gray matter, white matter, and cerebrospinal fluid components. MarsBaR toolbox with Automated Anatomical Labeling (AAL) template was employed to label the hippocampus and then their volumes were calculated.

**Results**

The distribution of hippocampus gray matter atrophy is strongly influenced by sex. Also the development and severity in the female patients with AD is much greater compared to male patients.

**Conclusions**

The study of AD based on the sex may help to figure out the root of AD mechanisms and potentially can be used as an imaging marker at early stages of study in the future.

**Acknowledgements**

This work was supported by project grants from Beijing Nova Program (xx2016120), National Natural Science Foundation of China (81101107), Natural Science Foundation of Beijing (4162008) and Young Innovative Talents of the Beijing Educational Committee (CIT & TCD201404053).

## 31 Atlas based dynamic functional connectivity of resting-state MEG data

### Yingnan Nie^1^, Chunlan Yang^1*^, Qun Wang^2^, Jiechuan Ren^2^, Wan Li^1^, Xin Zhang^1^

#### ^1^College of Life Science and Bioengineering, Beijing University of Technology, Beijing, China; ^2^Department of Internal Neurology, Tiantan Hospital, Beijing, China

##### **Correspondence:** Chunlan Yang (clyang@bjut.edu.cn)

**Aims**

This study seeks to develop an atlas-based beamformer approach of dynamic functional connectivity (FC) for resting-state MEG data, which is comparable with other imaging methods.

**Materials and methods**

The dynamic FC was calculated based on atlas with a sliding-window method. We first segmented the MEG data with a 2-seconds window, then performed a whole brain LCMV beamformer scan. The source with maximum power of each ROI was selected to compute the FC matrices. To avoid the problem of volume conduction, PLI was used to quantify the phase synchronization. Lastly, a k-means algorithm was applied to the windowed correlation matrices, each cluster centroid putatively reflects a stable FC state. We studied 16 healthy subjects of the Human Connectome Project (HCP) database.

**Results**

From the k-means analysis, we got 7 FC states. State 1 accounts for about 52% over all windows, which has the homologous pattern with the mean connectivity matrix. States 2–7 occur less frequently (ranging from 7.1% to 24.4%), but represent substantial different patterns with the mean connectivity.

**Conclusions**

In this work we proposed a novel framework to study the temporal variability of FC for resting-state MEG data through an atlas-based beamformer approach. Preliminary experimental results showed that even in the resting-state recording, FC changes among serval distinct states.

**Acknowledgements**

Supported by projects grant from Beijing Nova Program (XX2016120), National Natural Science Foundation of China (81101107) and Natural Science Foundation of Beijing (4162008).

## 32 Image quality assessment and enhancement of a thermal imager for photothermal therapy monitoring

### Fuwen Lai^1,2^, Mingwu Jin^1^

#### ^1^Department of Physics, University of Texas at Arlington, Arlington, TX 76051, USA; ^2^School of Instrument and Electronics, North University of China, Taiyuan, Shanxi, China

##### **Correspondence:** Mingwu Jin (mingwu@uta.edu)

**Aims**

This work is to investigate the spatial resolution and noise property of a thermal imager, FLIR C2, and to use this information to enhance the image quality of the thermal images for monitoring the millimeter-size treatment spot during a photothermal therapy.

**Materials and methods**

The slant-edge method was used to estimate the modulate transfer function (MTL) of FLIR C2 for the calculation of the point spread function (PSF). Three image enhancement methods were used to enhance the raw thermal images: 1) bi-lateral filtering (BF); 2) blind deconvolution with damping (BD); and 3) total-variation regularized deconvolution (TD) with PSF.

**Results**

The original spatial resolution of FLIR C2 is 0.37 cycles/mm (at 10% of MTF) and the noise is 0.53% at 23 °C and 0.60% at 50 °C. For test thermal images, TD achieved the best performance among three image enhancement methods on both edge recovery and noise suppression. Quantitatively, the TD method improves the spatial resolution of FLIR C2 to 0.68 cycles/mm and the noise slightly to 0.51% at 23 °C and 0.58% at 50 °C.

**Conclusions**

The TV-based method can significantly improve the resolution (84%) of FLIR C2 and enable temperature monitoring of the millimeter-size photothermal treatment spot with less than one percent variation.

**Acknowledgements**

Supported by a project grant from NIH 1R15CA199020-01A1.

## 33 Beamformed RF signals reconstruction in ultrasound imaging using sparse FFT

### Yifei Liu^1^, Mingyue Ding^2^, Yanhong Zhou^3^, Huihong Gong^1^, Wei Peng^1^

#### ^1^School of Biomedical Engineering, Hubei University of Science and Technology, Xianning, Hubei, 437100, China; ^2^College of Life Science and Technology, “Image Processing and Intelligence Control” Key Laboratory of Education Ministry of China, Huazhong University of Science and Technology, Wuhan, 437400, China; ^3^School of Medicine, Hubei University of Science and Technology, Xianning, Hubei, 437100, China.

##### **Correspondence:** Yifei Liu (yifeiliu@ymail.com)

**Aims**

Sparse fast Fourier transform (SFFT) is a class of sub-linear time algorithms for estimating the k largest frequency coefficients of a signal which is sparse in the frequency domain. As with the compressive sensing, it is possible for SFFT to acquire sparse signals far below the Nyquist rate, but SFFT is considerably simpler because the original signal can be simply restored by inverse fast Fourier transform. In addition, SFFT can get better reconstructed signal quality from fewer frequency coefficients. The purpose of this paper was to study the SFFT reconstruction for ultrasound beamformed signals.

**Materials and methods**

We used Field II ultrasound program with a linear array probe and cyst phantom to simulate the beamformed signals. The SFFT algorithms utilized 3% of signal length as the sparsity parameter k. A total of 50 rebuilt radio frequency (RF) scan lines were applied to assess the impact of SFFT reconstruction on associated beamformed image.

**Results**

The SFFT reconstruction quality was evaluated by keeping 10%-50% candidate frequency coefficients of the original beamformed signal. The results show SFFT can restore the normalized original beamformed scan lines with a mean absolute error range of [0.007-0.015]. The normalized root mean square error ranges for associated beamformed image is [0.052-0.095].

**Conclusions**

This paper shows the feasibility of SFFT for reconstructing the ultrasound beamformed signal. The future works include the reconstruction of raw channel RF signals and extending the study in 3D ultrasound imaging system.

## 34 Medical imaging application of improved genetic algorithm

### Tao Gong, Wenyu Liang

#### College of Information Science and Technology, Engr. Research Center of Digitized Textile & Fashion Tech. for Ministry of Education, Donghua University, Shanghai, 201620, China

##### **Correspondence:** Tao Gong (taogong@dhu.edu.cn)

**Aims**

In order to solve the slow convergence and early mature problems in searching for the image threshold with the traditional genetic algorithm and Otsu method, the genetic algorithm was improved for better image segmentation in the medical imaging of the lungs.

**Materials and methods**

According to the different evolution generation and the individual fitness, this improved genetic algorithm adjusted the strategies of elite selection and genetic operator dynamically, so it not only can speed up the convergence and the diversity of community, but also can get the best image segmentation threshold finally in a stable range. We used the dynamic crossover probability with the formula ${p}_c=\frac{k_1}{1+{e}^{\alpha \times gen}}+\lambda$ , and we set the parameters with *k*_1_ = 1, *α* = 0.055, *λ* = 0.3.

**Results**

The simulations were implemented with Matlab and some medical images of the lungs. The lung images showed that the left half pulmonary part was not healthy, the health condition of the right half part was better than the left one. Our approach got the better result and cost less computing time than both the simple Otsu method and the traditional genetic algorithm.

**Conclusions**

The proposed algorithm effectively segmented the original medical image, which made the target and background well separated. Our approach not only minimized the noise disturbance, but also enhanced the medical images of the left and right lungs, which was conducive to the subsequent medical diagnosis.

**Acknowledgements**

Supported by the project grants from National Natural Science Foundation of China (Grand No. 61673007, 61271114 and 61203325), Natural Science Foundation of Shanghai (Grand No. 13ZR1400200).

## 35 Combining graph cuts with improved Voronoi diagrams for the segmentation of overlapped cervical cells

### Lili Zhao^1^, Kuan Li^2^, Jianping Yin^3^_,_ Mao Wang^1^

#### ^1^College of Computer, National University of Defense Technology, Changsha, Hunan, 410073, China; ^2^Institute of Software, College of Computer, National University of Defense Technology, Changsha, Hunan, 410073, China; ^3^State Key Laboratory of High Performance Computing, National University of Defense Technology, Changsha, Hunan, 410073, China

##### **Correspondence:** Lili Zhao (yilinzhaohenan@126.com)

The segmentation of overlapped cervical cells from Pap smears is one of the most challenging problems in medical image processing field. We present a novel automated system for the cervical cell segmentation of cytoplasms and nuclei from multi-cell images. First, this system employed a graph cuts method including two algorithms, namely unsupervised k-means initialization and max-flow/min-cut optimization for scene segmentation. The segmented clumps were considered as foreground regions. Then, we used Voronoi diagrams to divide every clump of overlapping cells into individual non-overlapped regions, each containing one nucleus. Finally, to improve the overlapped segmentation, each individual cell in a clump was fitted by a minimum enclosing ellipse and the overlapped region was replaced by the corresponding area in this ellipse. This overlapped area and the connected free-lying region were combined to form a region of one complete cell. The experiments were conducted on two publicly released databsets downloaded from websites of University of Adelaide. The quantitative performance presents the average Dice Coefficient (DC) higher than 0.85. According to the explanation of evaluation metric in databsets, the “good” segmentation is evaluated with the DC > 0.7. Thus, the result of our proposed system outperforms 0.7 and achieves the state-of-art performance.

**Keywords**

Medical image processing, cervical smear image, overlapping segmentation, graph cuts, Voronoi diagrams.

**Acknowledgements**

Supported by the National Nature Science Foundation of China (Grant No. 61170287, 61232016, 61303189).

## 36 Intensity inhomogeneity correction algorithm for brain MRI image segmentation

### Wei Liu^1^, ZhiJun Gao^2^, LiSha Tan^3^

#### ^1^Network Center of Shenyang Jianzhu University, Liaoning, China; ^2^Graduate School of Shenyang Jianzhu University, Liaoning, China; ^3^Students' Affairs Division of Shenyang Jianzhu University, Liaoning, China

##### **Correspondence:** ZhiJun Gao (gzjun@sjzu.edu.cn)

**Aims**

To overcome the difficulty in accurate segmentation of brain magnetic resonance imaging (MRI), and reduce noise, partial volume effect and intensity inhomogeneity in MRI images.

**Materials and methods**

A brain MRI image segmentation strategy considering the intensity inhomogeneity is discussed in this paper. We improve basic fuzzy C-means clustering algorithm and propose a new algorithm using anisotropic diffusion for image segmentation. The correction of intensity inhomogeneity is also studied to be implemented to the actual work of MRI image segmentation.

**Results**

The improved algorithm has better restraint effect on intensity inhomogeneity and noise in brain MRI images, so the segmentation accuracy is enhanced. It can also well estimate the information of intensity inhomogeneity.

**Conclusions**

Our scheme can effectively estimate the intensity inhomogeneity information of images. We can get clearer segmentation images by removing intensity inhomogeneity, using the estimation of intensity inhomogeneity when performing image segmentation.

## 37 A voxel-based approach for sulci detection in 3D brain MR images

### Ke Gan, Daisheng Luo

#### College of Electronics and Information Engineering, Sichuan University, Chengdu, China

##### **Correspondence:** Ke Gan (gankeonline@hotmail.com)

**Aims**

This paper describes a fully automated method for sulci detection in 3D Magnetic Resonance (MR) images of human brain.

**Materials and methods**

To detect the brain sulci in 3D MR images, several consecutive steps were adopted. Brain image was automated segmented into white matter, gray matter and cerebrospinal fluid (CSF). A topological correction was carried out to make sure there is no direct contact between white matter and CSF. The gray matter/white matter boundary was extracted, and the Euclidean distance transformation was calculated from the boundary to the external regions. The gradient vector field of the distance map was calculated and diffused. Based on the diffused vector field, a divergence map was calculated. Candidate locations for the brain sulci were extracted from the divergence map by a thresholding procedure. Finally, morphological correction, including 3D connected component analysis and morphological operations, were applied to refine the detection results.

**Results**

This algorithm was implemented in C++ on a Windows platform and applied to label the brain sulci in 10 T1-weighted 3D brain MR images. Visual inspection indicates brain sulci in all testing images are correctly identified. Additionally, two major sulci (precentral sulcus and postcentral sulcus) in each hemisphere of the brain were manually labeled by a trained expert. And the overlap ratio between the manual labeling and the results of automated detection was calculated. Six imaging slices were selected from each testing image for the comparison. The average overlap ratio is 0.987±0.021 (the standard deviation).

**Conclusions**

The experimental results indicate this approach can be applied to extract the brain sulci in volumetric brain MR images.

## 38 CT angiography study on the configuration of the circle of Willis and measurement of posterior communicating artery

### Shaoyin Duan, Simin Lin, Hua Zhong, Shaomao Lv

#### Medical Imaging Department, Zhongshan Hospital of Xiamen University, Xiamen 361004, China

##### **Correspondence:** Shaoyin Duan (xmdsy@xmu.edu.cn)

**Aims**

To observe the configuration of the circle of Willis (CW), measure the size of posterior communicating artery (PcomA).

**Materials and methods**

73 cases CTA data of head and neck (without any lesion in the CW and its surrounding structures) were selected from our hospital. 3D images of CW were obtained, then CW was observed as two classifications and PcomA were measured.

**Results**

In 173 cases, classification 1 of CW found type I-IV is 41 cases (23.7% ), 103 (59.5%), 4 (59.5%) and 25 (14.5%), respectively; classification 2 found type I-IV is 26 cases (15%), 11 (6.4%), 116 (67.1%) and 20 (11.5%), respectively. Bilateral PcomA were showed in 45 cases with diameter of 128 ± 0.34mm, and the maximum is 1.8mm; the unilateral was showed in 36 cases with diameter of 1.14 ± 0.36 mm, and maximum 2.1mm; it is not significant difference of PcomA diameter between unilateral and bilateral, the left and right; no PcomA was showed in 92 cases.

**Conclusion**

Most of CW is the types of complete anterior circulation with incomplete posterior or developmental dysplasia. Diameter of PcomA is 1-2mm, and no PcomA is 53.18%.

**Acknowledgements**

Supported by the project grant from Natural Science Foundation of Fujian Province (Grand No. 2015J01535).

## 39 Early diagnosis of Parkinson disease via multimodal data

### Haijun Lei^1^, Jian Zhang^1^, Zhang Yang^2^, Baiying Lei^3^

#### ^1^College of Computer Science and Software Engineering, Shenzhen University, Key Laboratory of Service Computing and Applications, Guangdong Province Key Laboratory of Popular High Performance Computers, Shenzhen, China; ^2^School of Information Engineering, Shenzhen University, Shenzhen, China; ^3^School of Biomedical Engineering, Shenzhen University, National-Regional Key Technology Engineering Laboratory for Medical Ultrasound, Guangdong Key Laboratory for Biomedical Measurements and Ultrasound Imaging, Shenzhen, China

##### **Correspondence:** Baiying Lei (leiby@szu.edu.cn)

In this study, we proposed a new united features selection framework based on improved loss function to simultaneously perform classification and clinical sores prediction in Parkinson diseases via multi-modality neuroimaging data. The goal of the new united features selection model is to capture discriminative features that are used to train support vector regression model for clinical scores (sleep scores and olfactory scores) prediction and support vector classification model for class label identification. A promising classification and prediction performance was achieved on a dataset of 179 subjects (56 NC, 123 PD), with a 10-fold cross-validation. The experiment results demonstrated that, compared to only employ one single modality, multi-modality with MRI and DTI can effectively improve the performance in Parkinson classification. Compared to the state-of-art methods, the proposed method achieves a better performance in terms of disease status identification and clinical scores prediction.

**Keywords**: Parkinson’s disease, Feature selection, Classification, Prediction, Multi-modality

